# Static and dynamic bending has minor effects on xylem hydraulics of conifer branches (Picea abies, Pinus sylvestris)

**DOI:** 10.1111/pce.12307

**Published:** 2014-03-20

**Authors:** Stefan Mayr, Clara Bertel, Birgit Dämon, Barbara Beikircher

**Affiliations:** Department of Botany, University of InnsbruckSternwartestr. 15, A-6020, Innsbruck, Austria

**Keywords:** hydraulic efficiency, hydraulics, hydraulic safety, mechanics, vulnerability to embolism

## Abstract

The xylem hydraulic efficiency and safety is usually measured on mechanically unstressed samples, although trees may be exposed to combined hydraulic and mechanical stress in the field. We analysed changes in hydraulic conductivity and vulnerability to drought-induced embolism during static bending of *Picea abies* and *Pinus sylvestris* branches as well as the effect of dynamic bending on the vulnerability. We hypothesized this mechanical stress to substantially impair xylem hydraulics. Intense static bending caused an only small decrease in hydraulic conductance (−19.5 ± 2.4% in *P. abies*) but no shift in vulnerability thresholds. Dynamic bending caused a 0.4 and 0.8 MPa decrease of the water potential at 50 and 88% loss of conductivity in *P. sylvestris*, but did not affect vulnerability thresholds in *P. abies*. With respect to applied extreme bending radii, effects on plant hydraulics were surprisingly small and are thus probably of minor eco-physiological importance. More importantly, results indicate that available xylem hydraulic analyses (of conifers) sufficiently reflect plant hydraulics under field conditions.

## Introduction

Tree life is based on the formation of wood, which fulfils manifold functions. Metabolic processes and storage pools are provided by parenchyma cells, while dead cells with lignified cell walls facilitate the formation of complex axis systems. The latter enable trees to withstand gravitational and dynamic forces and to transport water into the crown. In angiosperms, the hydraulic function is provided by vessels, while fibre cells withstand self-loading and compression as well as tension forces. In contrast, tracheids fulfil both hydraulic and mechanical function in conifer xylem. Angiosperm and conifer conduits are connected by countless pits and are thus part of a complex transport network within the wood (Tyree & Zimmermann 2002).

Water transport in this conduit network is passively driven by tension (negative water potential Ψ) induced by transpiration and transmitted by continuous water columns to roots and soil (Boehm 1893; Dixon & Joly 1894). Under transpiration, transport resistances cause a decrease in Ψ from the roots towards the leaves. Plants can avoid steep Ψ gradients by stomata closure (at the cost of photosynthesis) and by formation of transport networks with low resistance (Tyree & Ewers 1991; Hacke & Sperry 2001). A so-called high hydraulic efficiency can be achieved, for example, by wide conduits and low pit resistances, and varies within trees to optimize their hydraulic architecture (Gartner 1995). Besides hydraulic efficiency, tree xylem also requires sufficient hydraulic safety: water columns in the xylem can be disrupted by the formation of gas bubbles in the xylem (i.e. embolism), which blocks the transduction of tensions and, in consequence, xylem water transport (Sperry & Tyree 1990; Tyree *et al*. 1994). Embolism can be induced by low Ψ, for example, during transpiration or in drought periods (Sperry & Tyree 1990; Choat *et al*. 2012), and by freeze-thaw events (Pittermann & Sperry 2003; Mayr & Sperry 2010). Xylem structures are therefore also optimized for embolism avoidance, for example, by efficiently sealing conifer pits, which block air entry into functional conduits (Delzon *et al*. 2010; Jansen *et al*. 2012). A balanced hydraulic efficiency and safety is a prerequisite for life and survival of plants (e.g. Tyree & Ewers 1991; Tyree & Zimmermann 2002; Beikircher & Mayr 2008), and avoidance of critical Ψ and embolism thus a main strategy, especially of woody plants (Choat *et al*. 2012). During the last decades, numerous studies focused on the vulnerability to embolism formation and its role in drought resistance.

There are many methods for vulnerability analysis available (e.g. Sperry *et al*. 1988; Cochard 1992; Alder *et al*. 1997; Cochard 2002; Li *et al*. 2008; Beikircher *et al*. 2010), whereby most of them are destructive and have to be performed in the laboratory. Typically, branches or stems of young trees are cut in the field and transported to the laboratory. Then, hydraulic analyses are performed under controlled conditions including avoidance of mechanical perturbation, such as bending of branches. Although avoidance of mechanical stress is important to enable accurate measures, this may limit the significance of hydraulic measurements for the interpretation of field situations. *In situ*, axes xylem is often exposed to combinatorial stresses and has to withstand various mechanical forces during drought or freeze-thaw stress. For instance, wind may cause enormous dynamic loads, and snow (Cannell & Morgan 1989), sand, litter or fruits large static bending loads (Niklas 1992; Mattheck 1996).

Mechanical stress may have consequences for xylem hydraulics at two levels. Firstly, plants react (in the long term) on mechanical stress by changes in xylem structures (Telewski 2006), which, in consequence, also influence hydraulic traits. For instance, Christensen-Dalsgaard *et al*. (2007) and Christensen-Dalsgaard *et al*. (2008) found a negative correlation between mechanical strain and the specific hydraulic conductivity (*k*_s_) within tropical trees. Spicer & Gartner (1998, 2002) demonstrated a decrease in hydraulic efficiency when formation of compression wood was induced by static bending of *Pseudotsuga menziesii*. Dynamic mechanical stress by repeated flexing of stems also caused a decrease in *k*_s_ of *Populus* stems (Kern *et al*. 2005). Furthermore, the hydraulic safety can be affected by mechanical adjustments during wood formation, as conifer compression wood was found to be more vulnerable to drought-induced embolism than opposite wood (Mayr & Cochard 2003). Secondly, there might also be instantaneous consequences of mechanical stress on the water columns and/or xylem structure, but only few studies dealt with this aspect. Han *et al*. (2007) reported a change in hydraulic conductance after bending of *Malus* stems in winter, which thus was not related to growth reactions of plants. In *Pinus taeda* stems bent to the point of imminent failure, an approximately 30% decrease in conductive cross-sectional area and a (not significant) decrease in sapwood conductivity after deflection were found (Fredericksen *et al*. 1994). Liu *et al*. (2003) reported a decrease in stem *k*_s_ of *Pinus contorta* trees, which were exposed to increased wind sway. In contrast, Christensen-Dalsgaard & Tyree (2013) analysed *Malus* and *Populus* stems, which were exposed to dynamic bending when frozen. The authors found no effect on percent loss of conductivity (PLC) and vulnerability to embolism when thawed samples were measured after the bending/freezing treatment. Please note that in all studies mentioned, hydraulic parameters were analysed after exposure to mechanical stress while an analysis of changes in hydraulic efficiency and safety at the time of mechanical stress, to our knowledge, is lacking.

The transport system may be instantaneously susceptible to bending stress for the following reasons. (1) Bending exposes the xylem to tensile and compressive forces leading to deformation of conduits. Changes in conduit shape may affect the hydraulic conductivity as it changes with the fourth power of the conduit radius (Tyree & Zimmermann 2002). In addition, deformation of pit structures may influence flow intensities, for example, when pit membrane pores are stretched and enlarged. (2) Bending causes compressive and tension forces acting on the cell wall in addition to forces caused by the negative Ψ in water columns. This combined stress may cause cell wall collapse as observed in conifer needle xylem (Cochard *et al*. 2004). (3) Pits are delicate structures within the cell wall and critical for air seeding (Tyree & Zimmermann 2002). Deformation of pit chambers may thus lead to air seeding and induce embolism. In the case of conifers, pit deformation may cause complete displacement of the torus, seal capillary seeding or torus capillary seeding by enlargement of pores within the torus (Tyree *et al*. 1994; Hacke & Sperry 2001; Domec *et al*. 2006; Cochard *et al*. 2009; Hacke & Jansen 2009; Delzon *et al*. 2010; Jansen *et al*. 2012). In addition, stretching of cell walls may enlarge cell wall pores and cause additional locations for air seeding (Domec *et al*. 2007). (4) Bending of branches may also cause local changes in tension, as water columns are compressed and stretched, especially under dynamic bending conditions. An increase in tension may be critical for embolism formation when xylem Ψ is already close to vulnerability thresholds (Choat *et al*. 2012).

In the present study, we therefore tested the direct effects of static bending on the hydraulic conductivity and direct effects of static and dynamic bending on the vulnerability to drought-induced embolism of two conifer species (*Picea abies*, *Pinus sylvestris*). We chose conifers because of the close link between hydraulic and mechanical function in their tracheid system (see above). We hypothesized that mechanical stress would affect xylem structures and/or the water column and thus result in reduced hydraulic efficiency and safety.

## Material and Methods

### Material

In a mixed conifer forest in Natters, Austria (11°21'E, 47°14'N, 838 m), south-exposed branches of Norway spruce (*P. abies* L. Karst) and Scots pine (*P. sylvestris* L.) were harvested, wrapped in plastic bags and transported to the laboratory. Branches were re-cut under water and rehydrated for at least 24 h. Up to 25-cm-long segments of the main shoot with a mean diameter of 7.3 ± 0.6 mm (*P. abies*) and 7.3 ± 0.5 mm (*P. sylvestris*) were used for conductivity measurements during static bending. For vulnerability analyses (static and dynamic bending), intact branches with a basal diameter of up to 1.5 cm and a length of up to 120 cm were taken.

### Bending experiments

**Figure 1:**
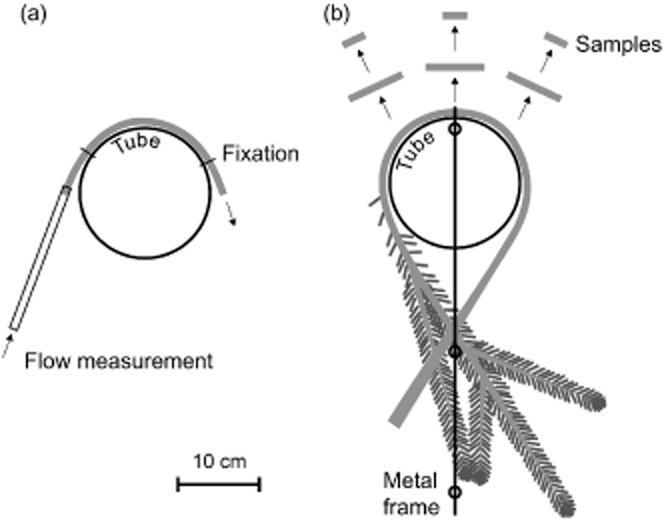
Static bending for conductivity and vulnerability analysis. (a) Samples for conductivity measurements were bent around plastic tubes of 80, 62.5 and 55 mm radius, fixed with wire slings and connected to the hydraulic measurement system with silicone tubes. (b) Branches for vulnerability analysis were bent around a tube of 80 mm radius and branch base and tip fixed with clamps on a metal frame. Samples for PLC measurements were taken from the central bent part of the main stem.

Static bending for conductivity measurements (hydraulic efficiency) was performed with plastic tubes with radii of 80, 62.5 and 55 mm (Fig. 1a). Fully hydrated samples were first measured without bending and then bent around the largest tube via wire slings for a second conductivity measurement. Subsequently, the samples were bent around the middle and smallest tube and measured. After measurements at 55 mm bending radius, samples were unbent and re-measured.

Static bending for vulnerability analysis (hydraulic safety) was performed with a tube of 80 mm radius, mounted to a metal frame. Central sections of branches were bent around the tube and branch base and tip fixed on the frame with clamps (Fig. 1b). Branches were then dehydrated to various extents at the bench (for 1 week at maximum). As a control for time effects (branches dehydrated longer were also exposed to longer bending), fully hydrated or slightly dehydrated branches were wrapped in plastic bags and bent for up to 7 d without dehydration.

For dynamic bending, a metal holder allowing bending of five branches in parallel was constructed. The base of branches was fixed in metal tubes and branches connected to a lever arm (in its initial position closest to branches) with a chain. The chain was fixed on branches with a cable strap whereby branches were protected by a foam sleeve. The lever was moved forward and backward by a wiper (VW Golf back window, Volkswagen, Germany) powered by a car battery. Branches were bent about 39 times per minute. The maximum deflection radius was about 67 cm whereby no bending stress acted on branches when the wiper arm reached its initial position. Branches were dehydrated to various extents while bending (for 1 week at maximum). As a control for time effects (branches dehydrated longer were moved over a longer period), some fully hydrated or slightly dehydrated branches were wrapped in plastic bags and moved for up to 7 d without dehydration.

Bending of branches was always achieved according to their position in the field. Therefore, the compression wood side (visible at the basal cut surface) was oriented to the centre of the bending circle. It was not possible to analyse the hydraulic efficiency during dynamic bending as required branch length would not allow measureable flow rates and movement of branches would cause biased flow patterns.

### Hydraulic conductance

The sample ends were debarked (about 2 cm at both ends) and re-cut under water several times prior to the experiment (Fig. 1a). The basal sample end was sealed into a silicone tube connected to the hydraulic system of a Xyl'em apparatus (Bronkhorst, Montigny les Cormeilles, France) filled with distilled, filtered (0.22 *μ*m) and degassed water containing 0.005% (v/v) ‘Micropur Forte MF 1000F’ (Katadyn Products Inc., Wallisellen, Switzerland) to prevent microbial growth (Beikircher & Mayr 2008). The distal end was submerged in water. The flow at a Δ*P* of 5–8 kPa then was determined before bending, during stepwise reduction of the bending radius and after release of the bending stress. The hydraulic conductance was calculated as percentage of initial conductance for each sample, respectively. After each bending step, flow rates reached a constant level within less than 1 min and were then taken for calculations.

### Vulnerability to drought-induced embolism, specific hydraulic conductivity

The water potential (Ψ) of branches from static or dynamic bending experiments was determined on up to 10-cm-long end twigs with a Scholander apparatus (Model 1000 Pressure Chamber; PMS Instrument Company, Corvallis, OR, USA). Three twigs per branch were measured and Ψs averaged.

**Figure 2:**
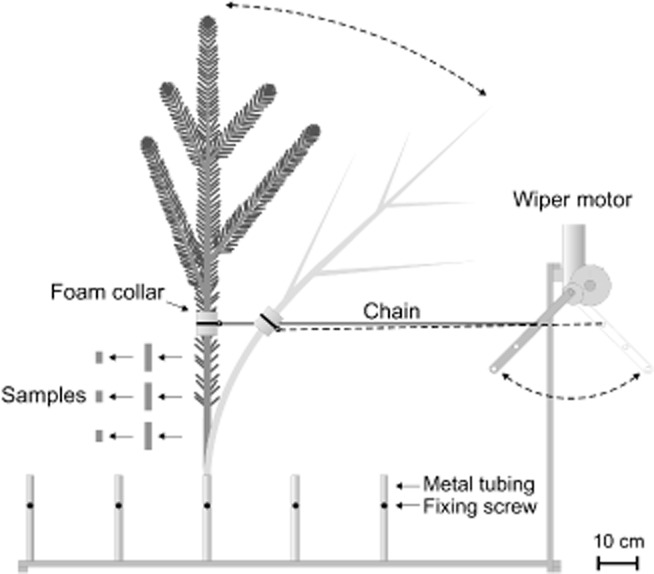
Dynamic bending for vulnerability analysis. Branches, fixed in metal tubes, were connected to a lever arm, which moved forward and backward 39 times per minute. Maximum deflection radius of branches was about 67 cm.

PLC of branches was quantified by measuring the increase in hydraulic conductance after removal of xylem embolism by repeated high pressure flushes (Sperry *et al*. 1988). Three samples from the bent stem section (Figs 1b and 2), up to 4 cm in length, were prepared as described in Mayr *et al*. (2002). Conductance measurements (at approximately 4 kPa) and flushing (at 80 kPa) were done with a Xyl'em apparatus using distilled, filtered (0.22 *μ*m) and degassed water containing 0.005% (v/v) ‘Micropur’ (see above). Flushing was repeated until measurements showed no further increase in conductivity. PLC was calculated from the ratio of initial to maximal conductance (Sperry *et al*. 1988) and values averaged per branch.

Vulnerability curves (PLC versus Ψ) were fitted with an exponential sigmoidal equation (Pammenter & Vander Willigen 1998):


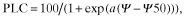
1

where PLC is the loss of conductivity (%), Ψ is the corresponding water potential, and *a* is a constant related to the curve slope. Ψ50 corresponds to Ψ at 50% PLC. Data (average PLC versus average Ψ of each branch) of treatments were pooled for construction of vulnerability curves. Curves were calculated using Fig.P 2.98 software (Biosoft, Cambridge, UK). Ψ at 12 and 88% PLC (Ψ12, Ψ88) were calculated from Ψ50 and parameter *a*, calculated standard errors refer to the regression fitting.

From maximum flow rates of unbent samples, we also calculated the specific hydraulic conductivity (*k*_s_):


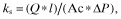
2

where *Q* is the volume flow rate (m^3^ s^−1^), *l* is the segment length (m), Ac is the xylem cross-sectional area (sapwood less heartwood; m^2^), and Δ*P* is the pressure difference between the segment ends (Pa). As flushing of samples with high PLC sometimes cannot completely restore the maximum conductivity, only samples with PLC < 60% were used for *k*_s_ determination. Measurements were corrected to 20 °C to account for changes in fluid viscosity with temperature.

### Number of samples and statistics

Conductivity measurements were performed on six branches per species. Vulnerability analysis was based on up to 27 branches per species and treatment, respectively. For control measurements, six to seven (static bending) and three (dynamic bending) branches were used per species. Results of conductivity measurements and vulnerability parameters are given as mean ± SE. Differences in conductivity were tested with paired Student's *t*-test after testing for Gaussian distribution (Kolmogorov–Smirnov test) and variance homogeneity (Levene test) of data. Vulnerability parameters were tested with the Welch's test. All tests were performed pairwise at a probability level of 5%.

## Results

### Bending stress

Applied bending stress intensities were high and in some cases exceeded the mechanical resilience of branches, which broke during experiments. These branches were excluded from further measurements and analyses. The maximum force (depending on the size and elasticity of branches) for static and elastic bending of branches was 10–20 N and 8.5–18.5 N per branch, respectively. For dynamic bending, the branch was moved from its initial to maximally bent position in about 0.77 s. The deflection was 18 cm and thus approximately 34.6% (related to the approximately 52-cm-long branch section, which was bent), which gives a deflection rate of approximately 45% s^−1^.

### Hydraulic conductivity, static bending

Branches of *P. abies* had slightly lower *k*_s_ (9.3 ± 1.0.10^−4^ cm^2^ s^−1^ MPa^−1^) than *P. sylvestris* (10.1 ± 1.1.10^−4^ cm^2^ s^−1^ MPa^−1^). In both species, bending caused a decrease in hydraulic conductance, with *P. abies* showing a more pronounced effect (Table 1). At a bending radius of 80 mm, the conductance decreased to about 90% and reached 80% at 55 mm radius. This effect was not related to a decrease in *k*_s_ over time as tested on unbent control samples, which were measured for 45 min (data not shown). *P. sylvestris* showed a similar trend but due to higher variation between samples, differences in conductance were not significant. When samples were unbent, the conductance increased but was overall 10% lower than before bending treatments (Table 1).

**Table 1:** Effects of static bending on the stem hydraulic conductance

	Bending			
(% of control)	*r* = 80 mm	*r* = 62.5 mm	*r* = 55 mm	After bending
*Picea abies*	90.8 ± 3.5^*^	83.9 ± 2.3^*^	80.5 ± 2.4^*^	87.7 ± 1.5^*^
*Pinus sylvestris*	97.6 ± 0.9	91.9 ± 1.8	89.0 ± 2.3	90.0 ± 2.3

Conductance of samples (related to initial conductance, % of control) after bending at different radii and release of bending stress (after bending). Stars indicate significant differences from unbent control at *P* ≤ 0.05. *n* = 6.

### Vulnerability to drought-induced embolism

**Figure 3:**
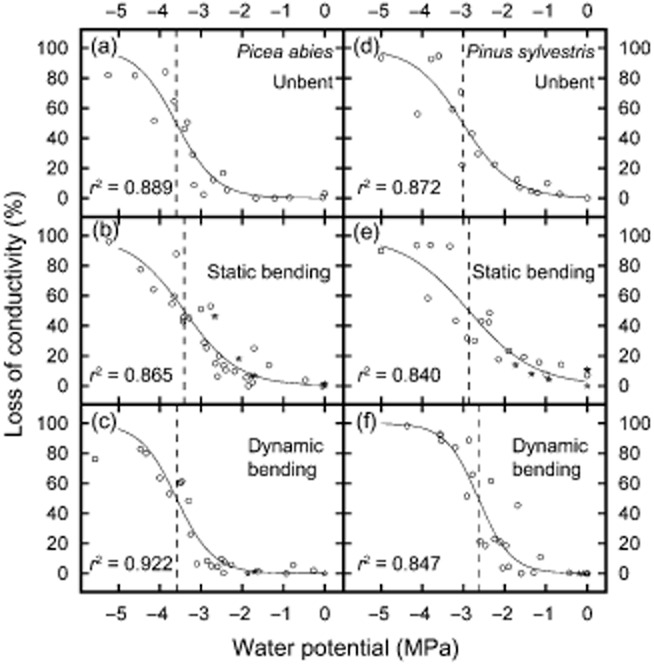
Vulnerability to drought-induced embolism. Vulnerability curves (PLC versus Ψ) of *Picea abies* (a–c) and *Pinus sylvestris* branches (d–f). Branches were either mechanically unstressed (a,d) or exposed to static (b,e) or dynamic bending (c,f) during dehydration. Vertical lines indicate Ψ50. Asterisks indicate controls (see Results).

Vulnerability analyses revealed a Ψ50 of −3.6 ± 0.1 MPa and −3.0 ± 0.1 MPa for *P. abies* and *P. sylvestris*, respectively (Fig. 3). In *P. abies*, neither static nor dynamic bending affected vulnerability to drought-induced embolism. In *P. sylvestris*, dynamic bending caused an approximately 0.4 and 0.8 MPa increase in Ψ50 and Ψ88, while static bending had no effect on vulnerability thresholds (Table 2). Control experiments showed that bending stress itself had no influence on PLC and that the duration of treatments thus did not bias vulnerability properties (Fig. 3).

**Table 2:** Effects of static and dynamic bending on the vulnerability to drought-induced embolism

	Ψ12 (MPa)	Ψ50 (MPa)	Ψ88 (MPa)	Parameter *a*
*Picea abies*				
Unbent	−2.63 ± 0.31	−3.60 ± 0.10	−4.57 ± 0.12	2.05 ± 0.43
Static bending	−2.10 ± 0.28	−3.41 ± 0.09	−4.72 ± 0.10	1.52 ± 0.22
Dynamic bending	−2.71 ± 0.21	−3.59 ± 0.07	−4.47 ± 0.07	2.26 ± 0.35
*Pinus sylvestris*				
Unbent	−1.84 ± 0.40	−3.02 ± 0.12	−4.19 ± 0.16	1.70 ± 0.38
Static bending	−1.27 ± 0.47	−2.87 ± 0.14	−4.48 ± 0.18	1.24 ± 0.24
Dynamic bending	−1.85 ± 0.24	−2.63 ± 0.08^*^	−3.43 ± 0.08^*^	2.52 ± 0.50

Water potential at 12, 50 and 88% of embolism (Ψ12, Ψ50, Ψ88) and parameter *a* of the vulnerability curve. Mean ± SE. Asterisks show comparisons in which values differ at *P* ≤ 0.05 from unbent controls. *n* = 19, 27, 22 (*P. abies*) and 18, 19, 24 (*P. sylvestris*) for unbent, static and dynamic bending, respectively.

## Discussion

Plant water transport occurs at negative Ψ and thus at a metastable state of water columns. The hydraulic efficiency and safety of the transport system are based on the xylem structures, which have to provide sufficient transport capacities as well as sufficient resistance to embolism formation. In conifers, all these functions are achieved by one cell type, the tracheid, which thus has to withstand also combinatorial stresses (Tyree & Zimmermann 2002).

Although there are multiple potential effects of mechanical stress on xylem hydraulics (see Introduction), we did not observe major implications upon bending of branches: the hydraulic efficiency was hardly affected by static bending. We observed a significant but small decrease (−20%) in conductance only in *P. abies* (Table 1). No significant effect on conductance was observed in *P. sylvestris*, which has more flexible stems. We also found an only small difference in conductance before and after bending, which is in accordance to the study on *P. taeda* in Fredericksen *et al*. (1994). Overall, the instantaneous effects of static bending on the hydraulic efficiency of conifer stems seem to be small, which indicates that responsible xylem structures undergo only minor hydraulically relevant deformation on bending. The small effects observed might be due to changes in conduit shapes and/or pit structures.

Surprisingly, we also found nearly no effect of static bending on the hydraulic safety (Table 2, Fig. 3). Vulnerability thresholds remained unaffected in *P. abies* and in *P. sylvestris*, which indicates that relevant structures, like the pits, were not substantially deformed and could maintain their function for hydraulic safety. Observed vulnerability thresholds thereby were similar to values published in other studies (e.g. Cochard 1992; Mayr *et al*. 2006).

Dynamic bending did not affect vulnerability thresholds in *P. abies*. Drought-induced PLC was similar to mechanically unstressed samples, even at low Ψ (Table 2, Fig. 3). In contrast, *P. sylvestris* showed higher vulnerability with increasing effects at lower Ψ. While Ψ12 was unaffected, Ψ50 and Ψ88 were shifted 0.4 and 0.8 MPa towards less negative Ψ. Differences between species are probably related to anatomical traits, such as conduit size and flexibility or pit structures. The observed effects on dynamic bending versus the lack of effects on static bending in *P. sylvestris* indicates that the movement of the branch is more critical than the bent status of a branch. Repeated bending might progressively weaken xylem structures or increase the probability that water columns or cell structures fail during one of the bending events.

Please note that branches in our experiments were exposed to high mechanical stress intensities with bending radii down to 55 mm (static bending of branches 7.3 mm in basal diameter) and 67 cm (dynamic bending 1.5 cm diameter). For example, Bründl *et al*. (1999) reported snow loads to cause a deflection of only 75–80 cm in approximately 200-cm-long spruce branches (diameter < 3 cm, snow load approximately 4 kg), which corresponds to a bending radius of 220–250 cm. High snow loads are known to cause damage (tip or branch breakages) in many trees, including *Picea* and *Pinus* species (Nykänen *et al*. 1997). Our bending treatment was clearly relatively close to the point of imminent failure as some branches broke (see Results). The frequency of experimental dynamic bending (1.5 s^−1^) was in the range of natural swaying of conifers. Rudnicki & Burns (2007) reported a natural sway frequency of 0.5 s^−1^ in *Pinus strobus* branches and Moore & Maguire (2004) showed a similar natural sway frequency of *P. abies* and *P. sylvestris* trees (0.39 and 0.35). Spatz *et al*. (2007) reported an eigenfrequency of approximately 4 s^−1^ in *P. menziesii* branches (0.6–1.6 m in length). However, within bending cycles of our dynamic experiments, deflection rates were high due to the strong bending. We reached a deflection rate of 45% s^−1^ (see Results) while, for example, Christensen-Dalsgaard & Tyree (2013) used a deflection rate of approximately 7% s^−1^ (1 cm deflection at 15 cm sample length). In conclusion, our experiments simulated high mechanical stress intensities. From an eco-physiological point of view, observed hydraulic effects might thus be of relevance only during extreme situations such as storm events or extreme winter precipitation. Moderate wind speeds or moderate snow loads will not affect the hydraulic efficiency and safety.

Probably more important is a methodical point of view: there are countless vulnerability analyses available (e.g. Cochard 1992; Alder *et al*. 1997; Martinez-Vilalta *et al*. 2004; Mayr *et al*. 2006; Li *et al*. 2008; Beikircher *et al*. 2010; Choat *et al*. 2012) that generally show the vulnerability of mechanically unstressed samples. The present study indicates, at least for conifers, that these vulnerability curves measured with available systems in the laboratory sufficiently reflect field situations. Although stems are exposed to various mechanical stresses in parallel to drought stress, the onset and intensity of embolism formation seems to be rather unaffected. The situation in angiosperms as well as the effects on freeze-thaw-induced embolism remains to be tested.

## Abbreviations

Ψ: water potential [Pa]
Ψ12, Ψ50, Ψ88: water potential at 12, 50 and 88% PLC [Pa]
*k*_s_: specific hydraulic conductivity [m^2^ s^−1^ Pa^−1^]
PLC: percent loss of conductivity [%]
